# Emotion regulation and drunkorexia behaviors among Lebanese adults: the indirect effects of positive and negative metacognition

**DOI:** 10.1186/s12888-022-04030-x

**Published:** 2022-06-10

**Authors:** Vanessa Azzi, Dora Bianchi, Sara Pompili, Fiorenzo Laghi, Sarah Gerges, Marwan Akel, Diana Malaeb, Sahar Obeid, Souheil Hallit

**Affiliations:** 1 School of Medicine and Medical Sciences, Holy Spirit University of Kaslik, P.O.Box 446, Jounieh, Lebanon; 2 Department of Developmental and Social Psychology, Sapienza University of Rome, Rome, Italy; 3 School of Pharmacy, Lebanese International University, Beirut, Lebanon; 4 School of Pharmacy, Gulf Medical University, Ajman, United Arab Emirates; 5 Social and Education Sciences Department, School of Arts and Sciences, Lebanese American University, Jbeil, Lebanon; 6 Psychology Department, College of Humanities, Effat University, Jeddah, 21478 Saudi Arabia; 7 Research Department, Psychiatric Hospital of the Cross, P.O. Box 60096, Jal Eddib, Lebanon

**Keywords:** Drunkrexia behaviors, Metacognition, Alcohol use disorder, Emotion regulation, Lebanese adults

## Abstract

**Background:**

Although metacognition processes are a core feature of restrictive eating and alcohol cravings and entail an individual to control both of his/her emotions and thoughts, yet, to our knowledge, a scarcity of research has examined their potential role in drunkorexia as cognitive and emotional predictors. The following study investigates the different associations between two emotion regulation strategies (i.e. emotional suppression and cognitive reappraisal) and drunkorexia behaviors in a sample of Lebanese adults, exploring the possible indirect effects of positive and negative alcohol-related metacognitions.

**Methods:**

This was a cross-sectional study that enrolled 335 participants (March-July 2021).

**Results:**

Higher problematic alcohol use (beta = 5.56), higher physical activity index (beta = 0.08), higher expressive suppression (beta = 0.23), higher negative metacognitive beliefs about cognitive harm due to drinking (beta = 0.75) and higher cognitive reappraisal (beta = 0.20) were significantly associated with more drunkorexic behaviors. The positive metacognitive beliefs about cognitive self-regulation significantly mediated the association between cognitive reappraisal and drunkorexia behaviors. Both the positive metacognitive beliefs about cognitive self-regulation and the negative metacognitive beliefs about the uncontrollability of drinking significantly mediated the association between expressive suppression and drunkorexia behaviors.

**Conclusion:**

This study demonstrated that emotional and metacognitive processes are associated with drunkorexia, addressing as well the mediating effect between deficient emotional regulation and risky behavioral patterns. Overall, our results would speculate that the lack of emotional and cognitive assets might enhance internal distress perceived out of control, leading individuals to indulge in maladaptive behavioral patterns for managing the underlying impairment.

## Introduction

Just over the last few years, popular media and researchers have denoted a particular interest in an upsurging trend: a problematic maladaptive behavior that has been alluded to as the non-medical term " drunkorexia," firstly presented by Chambers and CBS News in 2008 and afterward labeled "alcoholimia," a more medically comprehensive terminology [1, 2]. Although researchers have acknowledged the overlap between bulimic and anorexic tendencies in this behavioral pattern [3]; however, it displays specific prominent features with an ultimate association between binge drinking behaviors and caloric restriction [3]. Indeed, drunkorexia refers to indulging in a purposeful calorie restriction pattern on days when drinking alcohol is planned. The tendency to counterbalance the calories consumed through alcoholic beverages to prevent weight gain, together with the inclination towards enhancing the more intoxicating effects of alcohol, seem to be the fundamental motives underlying such behavior [4, 5]. In addition, several maladaptive behaviors have been reported to outreach the intended purposes, including fasting, skipping meals, using laxatives, self-induced vomiting, and strenuous exercising [6, 7].

This perilious pattern may severe negative consequences for physical and mental health: First, drunkorexia appears to be a risk factor for the establishment of cronic problem eating behaviors, such as binging and purging [8], and may lead to the subsequent onset of bulimia (1). Second, drunkorectic behaviors lead to more negative alcohol-related consequences: drunkeness, enhanced by calories restriction, may easily lead to memory blackouts, alcohol poisoning, and engagement in aggressive and risky behaviors [9–11]. Third, drunkorexia habits also are a risk factors for the development of alcohol abusive patterns and substance abuse [10, 12]. Available estimates have illustrated the high prevalence of drunkorexia in young adults, and specifically in college students [11, 13]. A recent Italian study [11] confirmed the upsurge of this perilous pattern among young adults, also revealing its association with alcohol and cocaine abuse. Regarding gender differences in drunkorexia behaviors, no differences were found in studies conducted among Lebanese [14] and among Italian adults [12]. Similar results were also confirmed in studies on adolescents [12].

Moreover, despite the overlap between dysfunctional eating behaviors and alcohol abuse that entail drunkorexia, it is still somewhat ambiguous whether the pattern is more associated with either one of these practices [15]. However, several researches have demonstrated that individuals' propensity to indulge in drunkorexia behavior is immensely enhanced by intensifying the intoxicating effects of alcohol [5]. Thus, it has been speculated that such disorder is more likely to be associated with problematic substance use [16]. Accordingly, another Italian study reported vastly high estimates of drunkorexia behavior among regular alcohol consumers compared to non-dependent users [17].

### Emotion regulation strategies and drunkorexia behaviors

Although potentially severe adverse outcomes have come in line with perilous weight management patterns and alcohol abuse, such as depression, anxiety, nutritional deficiencies, and cognitive dysfunction [11, 18], only a scarcity of research aimed to effectively delineate the intrinsic behavioral motives [19–21]. However, an emerging line of research is shedding light on the role of emotion dysregulation as one of the psychological triggers of drunkorexia patterns [14, 22]. Accordingly, the literature has emphasized the role of alcohol consumption as a behavioral strategy to regulate emotions: In absence of more adaptive cognitive strategies, alcohol consumption may promptly enhance positive affects while effectively altering the negative ones [23, 24].

Emotion regulation consists in the attempts to influence emotions through cognitive and behavioral strategies [25, 26]. According to the Process Model of Emotion Regulation [27], different strategies may be enacted in different moments during the emotion generation process: The antecedent-focused strategies modify the perception and interpretation of emotional cues at an early stage of this process, whereas response-focused strategies modify behavioral and physiological responses to an ongoing emotion [26, 27]. Two emotion regulation strategies have received major attention in research [28–30]: (1) cognitive reappraisal, which allows to interpret the meaning of a emotion-eliciting situation for changing its emotional impact (antecedent-focused); (2) expressive suppression, which allows to suppress or reduce the emotional expression at behavioral level (response-focused). These two strategies may have very different impacts on well-being [31, 32] in consideration of different aspects [26, 33]. Cognitive reappraisal has been often considered an adaptive strategy because it intervenes early in the process, altering the development of negative emotions and successfully reducing their impact on individual experience [28, 29]. Conversely, expressive suppression is generally considered maladaptive, as it enacts on the behavioral expression of emotions, but cannot avoid their inner experience: Individuals high in expressive suppression accumulate unresolved negative emotions and perceive a sense of incongruence between their feelings and behaviors, which may lead to psychological distress in the short and long term [31, 32].

On one hand, research on expressive suppression is quite consistent in addressing its negative consequences for well-being, including worse interpersonal functioning, more internalizing symptoms, and strong correlations with psychopatology, eating disorders and substance abuse [32–34]. On the other hand, research on cognitive reappraisal found weaker associations with psychological outcomes [31, 33], suggesting that contextual factors may someway shape the effects of this strategy on individual adjustment [26, 33]. For example, some studies suggested that adaptive strategies may lead to positive outcomes only when they are flexibly adopted in line with specific contextual demands [31–33].

In line with this research framework, the very limited findings conducted about emotion regulation strategies involved in drunkorexia [35] have revealed that young adults who partake in drunkorexia behaviors lacked one or many cognitive components related to emotion regulation, which would impede their adaptive strategies (i.e. cognitive reappraisal) to early manage negative emotions reducing their affective impact. Hence, drunkorexia behavior is presumed to be a coping approach more akin to expressive suppression, which can suppress intense emotional arousal, providing relief through alcohol misuse whenever other mechanisms of affect regulation are deficient [36]. Accordingly, there is initial evidence for a positive association of drunkorexia with expressive suppression strategies, while no relationships have been found with cognitive reappraisal [37]. Similarly, studies on problem drinking behaviors have found positive associations of alcohol abuse with expressive suppression, but no relationships with cognitive reappraisal [3, 38].

### Metacognition and drunkorexia behaviors

In line with this reasoning**,** it has also become compelling to thoroughly examine another innovative concept, a resolutive cognitive strategy that entails psychological features, apprehension, management, analysis, and modulation of an individual's cognition [39]. These cognitive processes have been labeled as "metacognition," a complex construct categorized according to positive or negative beliefs [40]. Precisely, positive metacognitions are speculated to appraise the functionality of cognitive processes as a means of directing and modulating thoughts and emotions. In contrast, negative metacognitive beliefs are conceptualized as a persuasion of lacking functional control over one's cognitions and affects [41]. According to the metacognition model [40], dysfunctional metacognitive processes may lead to maladaptive coping patterns, which can also involve problem drinking and eating behaviors [42].

As specifically regards the relationship of dysfunctional metacognition with alcohol use, researchers have enlightened the existence of specific alcohol-related metacognitive beliefs that encompass both positive evaluations about the effects of alcohol on cognitive processes (positive alcohol-related metacognitions, PAMS; e.g. “drinking allows me to control my thoughts and anxious feeling”), and negative beliefs about the alcohol-related cognitions (negative alcohol-related metacognitions, NAMS; e.g., “I cannot control drinking thoughts or behaviors”) [43]. Previous literature has highlighted an integral role of positive alcohol-related metacognitions in prompting individuals to engage in drinking behavior to manage their thoughts and emotions, as an adaptation strategy when negative thoughts are encountered. In contrast, negative beliefs contribute to maintaining such a pattern [44].

Similarly, other studies have established a direct association between eating disorders and dysfunctional metacognitive processes [45–47]. More precisely, individuals presenting eating disorders exhibit high levels of both positive metacognitions about the effectiveness of worrying thoughts, and negative beliefs, perceiving such thoughts as harmful and uncontrollable. Hence, they characterize the apprehension of gaining weight and preserving the shape found in eating disorders [48, 49] in addition to escalated alcohol cravings [50]. Moreover, several studies have highlighted the obsessive strive to control thoughts associated with eating disorders and drinking problems [51–53]. As such, individuals' propensity to engage in deleterious patterns reflects their tendency to manage their thoughts, avoid adverse outcomes, and compensate for their lack of effectively managing external circumstances or internal feelings; therefore, they opt for dysfunctional restrictive behaviors to perceive some self-control [54, 55]. Nevertheless, to date, only one study has established a direct relationship between metacognition and drunkorexia [56]: Specifically, drunkorexia behaviors were predicted by metacognitive beliefs about the need to control thoughts and about the uncontrollability and danger of thought processes, as well as by positive alcohol-related metacognitions.

### Relationships among metacognition, emotion regulation and drunkorexia behaviors

Both positive and negative metacognitive evaluations often entail emotional problems and emotion dysregulation, which may eventually contribute to psychological distress [57, 58]. In this regard, there is an emerging line of research which aims to understand the combined effects of metacognitive beliefs and emotion regulation in predicting psychological desease [59], as well as dysfunctional eating and drinking patterns [23, 60–62]. Specifically, Laghi et al. (2018) have found that the need to control thoughts (dysfunctional metacognive belief) moderated the association between the lack of emotional awareness (emotional difficulty) and problem eating behaviors [62]. Moreover, emotion regulation difficulties have been found to enhance positive alcohol-related metacognitive beliefs, which in turn lead to more alcohol misuse [23].

From this standpoint, previous literature speculated an integral motivational role of metacognitive beliefs in using alcohol and restricting eating behaviors as a cognitive-emotional regulation strategy [23]. It is presumed that since metacognition fulfills a cognitive rumination role, it also exhibits a fundamental emotion regulation function [42], hence contributing to a general emotional dysregulation. To specify, researchers found that negative cognitions are mobilized in alcohol dependence settings; such cognitions trigger adverse emotional distress that exacerbates alcohol cravings [63]. According to the speculated theory, emotional disorder entails underlying cognitive impairment, responsible for cravings escalation [64]. For instance, Dragan [23] interestingly found no direct relationship between emotional dysregulation and drinking behavior except when positive metacognitive beliefs were established as anticipated mediators. Indistinctly, positive and negative metacognitions compel the individual to engage in drinking behaviors (a core aspect of drunkorexia), adapt to the emotions and cognitions, and perpetuate such a process. Conversely, emotional dysregulation and impaired cognitions were both identified as predictors of eating disorders (another core component of drunkorexia). Namely, the strive to control thoughts, an integral function of metacognition, was significantly reported in deleterious eating behaviors [65]. Remarkably, the lack of modulating adverse emotions is also conceptualized as a form of loss of control [66]; hence compensatory behaviors are perceived as mandatory. In line with what has been previously stated, a Lebanese study hypothesized the potentially mediating role of metacognition in assessing the relationship between emotion regulation and drunkorexia [14].

Although metacognition processes are a core feature of restrictive eating and alcohol cravings yet, to our knowledge, there is a scarcity of research that has thoroughly examined their roles in drunkorexia as cognitive and emotional predictors. Hence, it is conceivable that the role of metacognitions may have been underscored in understanding drunkorexia patterns since it is quite likely that both cognitive and emotional states affect the potential to modulate one's behaviors. However, to our knowledge, no other study has so far examined the mediating role of metacognitive processes in the association between drunkorexia and emotion regulation.

### The current study

Based on the abovementioned research [10; 23], the present study aims to investigate the different associations between two emotion regulation strategies (i.e. emotional suppression and cognitive reappraisal) and drunkorexia behaviors in a sample of Lebanese adults, exploring the possible indirect effects of positive and negative alcohol-related metacognitions. In light of recent evidence about the associations between drunkorexia and emotion regulation [14, 37, 39, 67], we expect that only dysfunctional emotion regulation processes (i.e. expressive suppression) may be directly related to drunkorexia behaviors [37]. Conversely, in line with evidence that other variables may determine the positive vs. negative outcomes of supposed adaptive emotion regulation strategies (i.e. cognitive reappraisal) [31–33], we specifically hypothesized that cognitive reappraisal might be related to drunkorexia behaviors only via the indirect effect of alcohol-related metacognitions [39].

## Methods

### Study design and procedure

This was a cross-sectional study based on an online anonymous survey. It was conducted from March until July 2021. The voluntary survey was carried out on the Lebanese population located in all Governorates of Lebanon (Beirut, Mount Lebanon, North, South, and Bekaa). To minimize interview risks as well as the lockdown restrictions enforced by the Lebanese Government, a snowball sampling method was used as an approach to the survey using online Google forms. The survey was distributed via social applications including WhatsApp, LinkedIn, and Facebook. As previously documented in the literature, the research based on an online questionnaire creates the opportunity to collect data nationwide and reach specific groups of individuals [68, 69]. All invited participants were alcohol drinkers and above 18 years of age (information was self-reported by the participants).

### Minimal sample size calculation

Based on a correlation coefficient of 0.27 between positive metacognition and drunkorexia behaviors [56], and based on a risk of error α = 5% and power of 95%, the G-power software calculated a minimal sample size of 140 participants to ensure enough statistical power for the multivariable analysis.

### Questionnaire

The anonymous, self-administered questionnaire used was in Arabic, the native language of Lebanon, and required approximately 20 min to be completed. Participants were asked to fill out the questionnaire without the request of any help to avoid any potential influence when answering the questions.

The first part of the questionnaire evaluated participants' sociodemographic information (age; marital status, and educational level). Educational level was categorized into secondary or less and university level. In addition, the household crowding index was calculated by dividing the number of persons living in the house by the number of rooms, excluding the bathroom and the kitchen [70]. The physical activity index was computed by multiplying the physical activity’s strength by its frequency by its duration [71].

The second part of the questionnaire was composed of the different scales used:

- **Drunkorexia motives and behaviors scales (DMBS)**
  The DMBS contains a total of 52 items that evaluate participants’ engagement in drunkorexia [72]. Each item includes the prompt “Rate the frequency of each statement” and the items are on a Likert type-scale including never (1), seldom (2), sometimes (3), often (4), and very often (5). In the present study, only one dimension from the DMBS has been used: drunkorexia behaviors (8 items) that relate to different behaviors associated with drunkorexia (e.g., “By exercising more than normal”). The possible score ranged from a minimum of 8 to a maximum of 40. In our study, the mean score of drunkorexia behaviors (M = 10.93) was lower than means obtained in other international studies [50; 55]; but it was consistent with previous Lebanese studies conducted on adult samples [19]. This dimension also reached excellent reliability in this study (Cronbach’s *α* = 0.950).
- **Emotion regulation**
  Emotion regulation strategies were assessed with the Lebanese version [56] of the Emotion Regulation Questionnaire (ERQ) [34]. This scale is composed of 10 questions that are scored on a seven-point Likert scale (1 = strongly disagree to 7 = strongly agree). This scale yields two scores, the Cognitive Reappraisal (6 items; possible range: 6–42; e.g. “I control my emotions by changing the way I think about the situation I’m in”) and Expressive Suppression (4 items; possible range: 4–28; e.g. “I control my emotions by not expressing them”). Higher scores indicate more cognitive reappraisal and more expressive suppression respectively (Cronbach's alpha for cognitive reappraisal in this study = 0.881 and expressive suppression = 0.819). Also in this scale, mean scores obtained in our sample (30.21 for cognitive reappraisal; and 18.18 for expressive suppression) were similar to mean total scores detected in previous international studies on adults [73].
- **AUDIT scale**
  The self-reported ten-item scale, validated in Lebanon [74], was used to assess problematic alcohol use [75]. Problematic alcohol use was considered when participants scored 8 or more [75]. In the current sample, Cronbach's alpha for the total scale was 0.864.
- **Positive alcohol metacognitions scale (PAMS)**
  PAMS is a 12-item measure developed to assess positive metacognitions about alcohol use [43]. It consists of two factors: Factor 1: positive metacognition beliefs about emotional self-regulation (composed of 8 questions, e.g.: "Drinking reduces my anxious feelings"; score range: 8–32) and Factor 2: positive metacognitive beliefs about cognitive self-regulation (composed of 4 questions, e.g.: "Drinking helps me to control my thoughts"; score range: 4–16). All questions were scored on a four-point Likert scale (1 = strongly disagree to 4 = strongly agree). In the current sample, the Cronbach's alpha values were 0.918 for Factor 1 and 0.847 for Factor 2. Moreover, the mean score of Factor 1 in our study sample (19.24) was very similar to other international studies [43, 56], whilst the mean score in Factor 2 (7.25) was higher among Lebanese sample,in comparison with other studies [43, 56].
- **Negative alcohol metacognitions scale (NAMS)**
  NAMS is a 6-item measure developed to assess negative metacognitions about alcohol use [43]. It is composed of 2 factors: Factor 1: negative metacognitive beliefs about the uncontrollability of drinking (3 items, e.g.: "I have no control over my drinking"; score range: 3–12) and Factor 2: negative metacognitive beliefs about cognitive harm due to drinking (3 items, e.g.: "Drinking will damage my mind"; score range: 3–12). Items are rated on a 4-point Likert scale from 1 (Not Agree) to 4 (Agree very much). In the current sample, the Cronbach's alpha values were 0.885 for Factor 1 and 0.734 for Factor 2. Finally the mean scores emerged in our sample for Factor 1 (4.24) and for Factor 2 (5.22) were in accordance with mean scores obtained on these dimensions in previous international studies [59].

### Statistical analysis

Data analysis was performed on SPSS software version 25. The drunkorexia behaviors score followed a normal distribution, as verified by the skewness and kurtosis values (between -2 and + 2) [76]. These conditions consolidate the assumptions of normality in samples larger than 300 [77]. Pearson correlations were computed between drunkorexia and continuous variables of the study. The Student t-test and ANOVA tests were used instead for categorical variables, in order to compare drunkorexia mean scores in different groups. Effect sizes were calculated for all bivariate analyses; in psychological research, values of 0.1 were deemed to have a small effect size, whereas values of 0.2 and 0.3 were classified as having medium and large effect sizes respectively [78]. A forward linear regression was then conducted, entering sociodemographic variables, problematic alcohol use, emotion regulation strategies, and positive and negative alcohol-related metacognition scales as independent variables, and drunkorexia behavior as criterion variables.

The PROCESS SPSS Macro version 3.4 model four [79] was used to estimate the indirect effects of each emotion regulation strategy on drunkorexia behaviors via positive and negative alcohol-related metacognitions. Specifically, for each model three pathways were computed: Pathway A determined the regression coefficient for the effect of each emotion regulation strategy on Positive/Negative Alcohol Metacognitions Scales, Pathway B examined the association between Positive/Negative Alcohol Metacognitions Scales and drunkorexia behaviors, independent of emotion regulation, and Pathway C and C' estimated the total and the direct effects of emotion regulation strategies on drunkorexia behaviors. Pathway AB calculated the indirect intervention effects; specifically, different mediation models were tested, in which the indirect effects from each emotion regulation strategy on drunkorexia behaviors were computed via each metacognitive dimension. To test the significance of the indirect effects, the macro generated bias-corrected bootstrapped 95% confidence intervals (CI) [79]. A significant mediation was determined if the CI around the indirect effect did not include zero [79]. Independent variables entered in the final model were those that showed a correlation coefficient or an effect size > ׀ 0.24 ׀ to have more parsimonious models [80]. A *p* < 0.05 was considered significant.

## Results

### Sociodemographic and other characteristics of the participants

The mean age of the sample was 32.16 ± 11.09 years, with 52.5% males (age range: 18–66 years). The mean drunkorexia behaviors score was 10.93 ± 8.87. More details about the sample can be found in Table 1.

**Table 1:** Sociodemographic and other characteristics of the participants (*N* = 335)

Variable	N (%)
Gender	
Male	176 (52.5%)
Female	159 (47.5%)
Marital status	
Single	187 (55.8%)
Married	148 (44.2%)
Religion	
Christian	193 (57.6%)
Muslim	94 (28.1%)
Druze	48 (14.3%)
Education level	
Secondary or less	56 (16.7%)
University	279 (83.3%)
Problematic alcohol use (PAU)	
Low PAU (AUDIT scores of 7 or less)	232 (69.3%)
High PAU (AUDIT scores of 8 or more)	103 (30.7%)
	**Mean ± SD**
Age (in years)	32.16 ± 11.09
Number of children	0.95 ± 1.17
Physical activity index	27.81 ± 20.22
Household crowding index	0.97 ± 0.40
Body mass index (kg/m^2^)	24.16 ± 3.90
Drunkorexia behaviors	10.93 ± 8.87
Cognitive reappraisal	30.21 ± 7.79
Expressive suppression	18.18 ± 5.92
Positive metacognition beliefs about emotional self-regulation	19.24 ± 6.40
Positive metacognitive beliefs about cognitive self-regulation	7.29 ± 2.86
Negative metacognitive beliefs about the uncontrollability of drinking	4.24 ± 1.99
Negative metacognitive beliefs about cognitive harm due to drinking	5.22 ± 2.38

### Bivariate analysis

Bivariate analysis results are summarized in Tables 2 and 3. Participants with a secondary level of education or less and those with high problematic alcohol use had significantly higher mean drunkorexia behaviors scores. Furthermore, higher age, physical activity index, cognitive reappraisal, expressive suppression, positive metacognitive beliefs about cognitive self-regulation, negative metacognitive beliefs about the uncontrollability of drinking, and negative metacognitive beliefs about cognitive harm due to drinking were significantly associated with more drunkorexia behaviors.

**Table 2:** Bivariate analysis of continuous variables associated with drunkorexia behaviors

Variable	DB	Age	NC	PAI	HCI	BMI	CR	ES	PAMS1	PAMS2	NAMS1	NAMS2
Drunkorexic behaviors (DB)	1											
Age	.16^**^	1										
Number of children (NC)	.01	.68^***^	1									
Physical activity index (PAI)	.25^***^	-.15^**^	-.13^*^									
Household crowding index (HCI)	.02	-.08	.03	.02	1							
Body mass index (BMI)	.04	.32^***^	.24^***^	-.04	-.08	1						
Cognitive reappraisal (CR)	.25^***^	.20^***^	.08	.05	-.04	.04	1					
Expressive suppression (ES)	.33^***^	.06	-.05	.12^*^	.09	.08	.55^***^	1				
PAMS1	.08	.11^*^	.01	-.23^***^	.04	.11	.21^***^	.13^*^	1			
PAMS 2	.26^***^	.06	-.02	-.07	.12^*^	.01	.19^**^	.29^***^	.63^***^	1		
NAMS 1	.35^***^	.07	-.05	.01	.24^***^	.06	.04	.20^***^	.36^***^	.41^***^	1	
NAMS 2	.29^***^	.13^*^	.06	.02	.10	.04	.03	.12^*^	.18^**^	.21^***^	.48^***^	1

*R* Pearson correlation coefficient, ^***^p < 0.001; ^**^p < 0.01; ^*^p < 0.05; *PAMS1* Positive metacognition beliefs about emotional self-regulation, *PAMS2* Positive metacognitive beliefs about cognitive self-regulation, *NAMS1* Negative metacognitive beliefs about the uncontrollability of drinking, *NAMS2* Negative metacognitive beliefs about cognitive harm due to drinking

**Table 3:** Bivariate analysis of categorical variables associated with drunkorexia behaviors

Variable	Mean ± SD	*p*	Effect size
Gender		0.214	0.136
Male	10.35 ± 8.64		
Female	11.56 ± 9.09		
Marital status		0.488	0.075
Single	10.63 ± 8.85		
Married	11.30 ± 8.90		
Religion		0.233	0.093
Christian	11.20 ± 9.19		
Muslim	9.71 ± 8.58		
Druze	12.19 ± 7.92		
Education level		**0.003**	0.408
Secondary or less	13.71 ± 7.16		
University	10.37 ± 9.08		
Problematic alcohol use (PAU)		** < 0.001**	0.853
Low PAU (AUDIT scores of 7 or less)	8.79 ± 8.48		
High PAU (AUDIT scores of 8 or more)	15.74 ± 7.80		

### Multivariable analysis

Despite positive and negative alcohol-related metacognitions showing an effect size lower than the fixed value (0.24), we forced these subscales into the model, consistently with our research hypotheses. The results of stepwise linear regression, taking the drunkorexia behaviors score as the dependent variable, showed that higher problematic alcohol use (beta = 5.56), higher physical activity index (beta = 0.08), higher expressive suppression (beta = 0.23), higher negative metacognitive beliefs about cognitive harm due to drinking (beta = 0.75) and higher cognitive reappraisal (beta = 0.20) were significantly associated with more drunkorexia behaviors (Table 4). The model explained a significant 30.6% of the variance in drunkorexia behaviors.

**Table 4:** Multivariable analysis: Stepwise linear regression taking the drunkorexia behaviors score as the dependent variable

Variable	Unstandardized Beta	Standardized Beta	*p*	95% CI
Problematic alcohol use (high vs low^*^)	5.56	0.29	** < 0.001**	3.74–7.38
Expressive suppression	0.23	0.15	**0.006**	0.07–0.40
Negative metacognitive beliefs about cognitive harm due to drinking	0.75	0.20	** < 0.001**	0.41–1.10
Physical activity index	0.08	0.18	** < 0.001**	0.04–0.12
Cognitive reappraisal	0.20	0.18	**0.002**	0.08–0.33

^*^ Problematic alcohol use was coded as: Low = 0 and High = 1; numbers in bold indicate significant *p*-values

### Mediation analysis

**Fig. 1:**
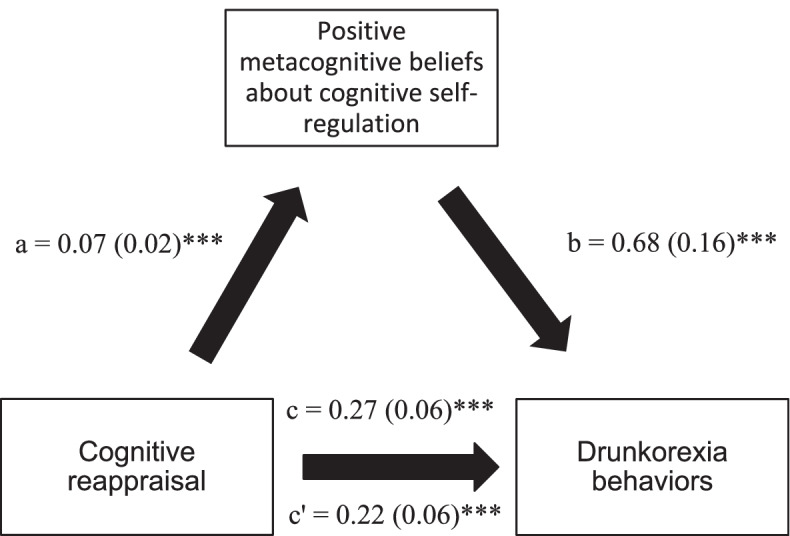
**a** Relation between cognitive reappraisal and positive metacognitive beliefs about cognitive self-regulation; **b** Relation between positive metacognitive beliefs about cognitive self-regulation and Drunkorexia behaviors; **c** total effect of cognitive reappraisal on drunkorexia behaviors; (c’) direct effect of emotion regulation on drunkorexia behaviors. Numbers are displayed as regression coefficients (standard error). *p < 0.05; **p < 0.01; ***p < 0.001

**Fig. 2:**
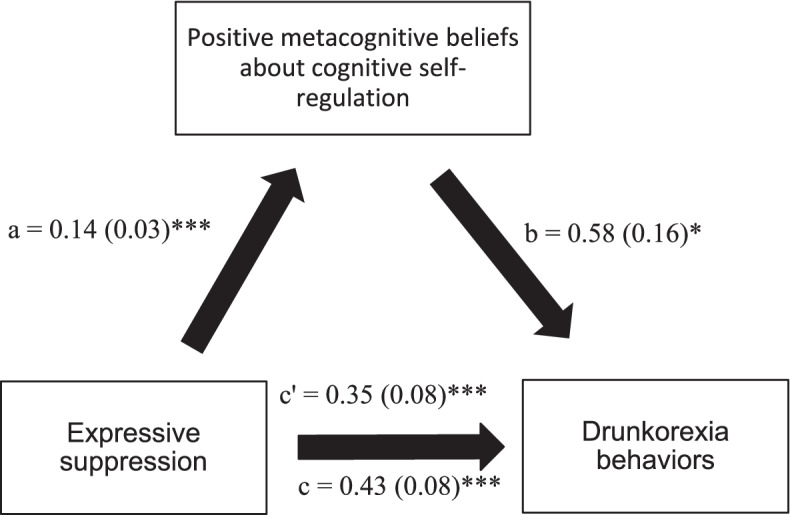
**a** Relation between expressive suppression and positive metacognitive beliefs about cognitive self-regulation; **b** Relation between positive metacognitive beliefs about cognitive self-regulation; **c** total effect of expressive suppression on drunkorexia behaviors; (c’) direct effect of emotion regulation on drunkorexia behaviors. Numbers are displayed as regression coefficients (standard error). *p = 0.01; **p = 0.001; ***p < 0.001

**Fig. 3:**
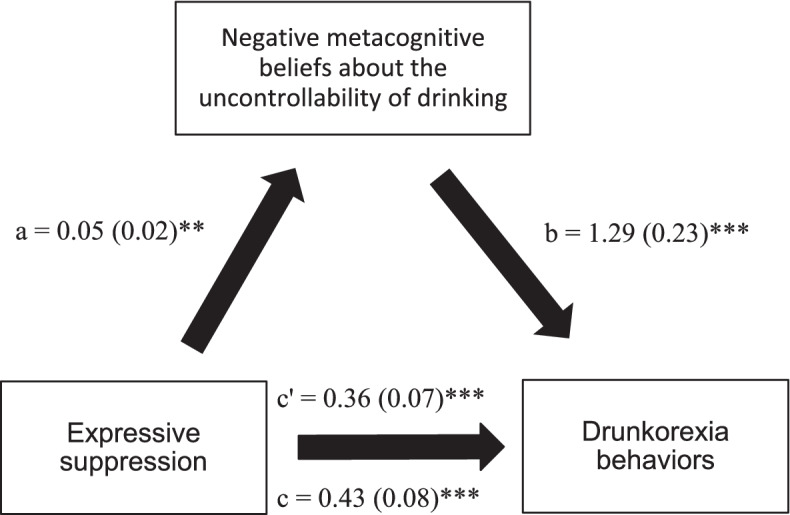
**a** Relation between expressive suppression and negative metacognitive beliefs about the uncontrollability of drinking; **b** Relation between negative metacognitive beliefs about the uncontrollability of drinking and drunkorexia behaviors; **c** total effect of expressive suppression on drunkorexia behaviors; (c’) direct effect of emotion regulation on drunkorexia behaviors. Numbers are displayed as regression coefficients (standard error). *p = 0.01; **p = 0.001; ***p < 0.001

The results of the mediation analysis are summarized in Table 5. The positive metacognitive beliefs about cognitive self-regulation significantly mediated the association between cognitive reappraisal and drunkorexia behaviors (Table 5, Model 1, and Fig. 1). Moreover, both the positive metacognitive beliefs about cognitive self-regulation and the negative metacognitive beliefs about the uncontrollability of drinking significantly mediated the association between expressive suppression and drunkorexia behaviors (Table 5, Model 2, and Figs. 2 and 3).

**Table 5:** Mediation analysis: Direct and indirect effects of the associations between emotion regulation strategies, positive and negative alcohol metacognitions subscales, and drunkorexia behaviors

Model 1: Cognitive reappraisal taken as independent variable						
**Mediator**	**Direct effect**			**Indirect effect**		
	**Effect**	**SE**	**p**	**Effect**	**SE**	**95% BCa**
PAMS Factor 1	0.25	0.06	< 0.001	0.02	0.02	-0.01–0.05
PAMS Factor 2	0.22	0.06	< 0.001	0.05	0.02	**0.01–0.08**
NAMS Factor 1	0.26	0.05	< 0.001	0.01	0.02	-0.03–0.05
NAMS Factor 2	0.26	0.06	< 0.001	0.01	0.02	-0.02—0.04
**Model 2: Expressive suppression taken as independent variable**						
**Mediator**	**Direct effect**			**Indirect effect**		
	**Effect**	**SE**	**p**	**Effect**	**SE**	**95% BCa**
PAMS Factor 1	0.41	0.08	< 0.001	0.02	0.02	-0.01—0.06
PAMS Factor 2	0.35	0.08	< 0.001	0.08	0.03	**0.03–0.15**
NAMS Factor 1	0.36	0.07	< 0.001	0.07	0.02	**0.03–0.12**
NAMS Factor 2	0.38	0.07	< 0.001	0.03	0.02	-0.01—0.07

Numbers in bold indicate significant mediation

Direct effect = effect of emotion regulation strategy on drunkorexia behaviors in the absence of the mediator; Indirect effect = Effect of the emotion regulation strategy on drunkorexia behaviors in the presence of the mediator; SE = Standard Error; BCa = Bootstrap Confidence Interval. Mediator in Model 1: Positive metacognition beliefs about emotional self-regulation; Mediator in Model 2: Positive metacognitive beliefs about cognitive self-regulation; Mediator in Model 3: Negative metacognitive beliefs about the uncontrollability of drinking. mediator in Model 4: Negative metacognitive beliefs about cognitive harm due to drinking

## Discussion

This study shed new light on the emotional and metacognitive correlates of drunkorexia in young adults, providing interesting insights into their direct and indirect associations.

In the first place, our findings revealed that both functional (cognitive reappraisal) and dysfunctional (expressive suppression) emotional regulation strategies are significantly associated with drunkorexia behavior. These findings are consistent with prior research about the scarcity of emotional competencies which leads adolescents to indulge in drunkorexia behaviors as a maladaptive coping strategy for managing emotional states [35]. Either adaptive or maladaptive emotion regulation strategies may however intervene when emotional competence fails in accepting and expressing ongoing emotions [21, 22]. In fact, our findings provide a first evidence for the association of both functional and dysfunctional emotion regulation processes with drunkorexia, which appears to be a possible behavioral outcome. In previous literature it has been observed that in view of palliating nervousness [81], assuaging emotional arousal, acquiring a sense of control over impulsiveness, thus, overall inhibiting and avoiding emotional experiences [39, 82, 83], young adults tend to adopt restrictive eating and drinking behaviors [84, 85]. Accordingly, "emotion regulate skills" empower an individual to recognize, monitor, assess, and adapt his/her emotional response as a way of achieving a goal-directed behavior most appropriately [30, 86]. Hence, this suggests that self-imposed inappropriate restrictive patterns may offer a sense of reassurance over a threatening situation or an aversive emotional state (e.g. self shape-criticism), thus, alleviating their inner anxiousness [87–89].

Moreover, Ward and Galante [72] speculated that those confronting aversive emotional states are more prone to indulge in risky behavioral drinking patterns whether to build up positive emotions, adhere to peers' expectations and approval, or alleviate their inner adverse affects. Likewise, several theorists have highlighted the deficient emotional competencies amongst those individuals who find themselves powerless in coping with intense emotional experiences and hence, indulge in deleterious patterns as a means of evasion and inhibition of intense affects [84].

In the second place, our research also revealed that dysfunctional metacognition, evaluated through positive and negative metacognitive beliefs about alcohol use, is strictly associated with drunkorexia behavior. These results can be explained in light of previous literature about metacognition in eating and drinking problem behaviors. For example, the need to control thoughts is a maladaptive metacognitive belief that constitutes a well-proven susceptibility factor for dysfunctional eating behaviors [90, 91]. Another metacognitive belief, low cognitive confidence, insinuates persistent apprehension of an individual's attention and impedes appropriate adaptive strategies for coping with distress, therefore enhancing worry and rumination, two major maladaptive metacognitive processes tightly allied to eating behaviors [65, 92]. Moreover, it has been conceived that perceptions and memories related to drinking behaviors set off positive metacognitive thoughts in the form of progressed rumination and worry; hence, enhancing cravings [92].

Conversely, Clark et al. [93] shed light on "metacognitive monitoring" englobing cognitive-emotional regulation, consciousness about personal aims, and their fulfillment strategy. Specifically, those who indulge in maladaptive eating behaviors exhibit an intense obsession with controlling and suppressing thoughts related to their shape, hence maintaining excessive attentiveness to these issues leading to the perpetuation of the disorder [62].

In this perspective, Laghi et al. [56] showed that positive metacognitive beliefs concerning alcohol provide individuals a more robust sense of control and monitoring over burdensome cognitions and emotions; therefore considered to be significant risky predictors of drunkorexia behavior [23, 53].

Interestingly, the Italian study [56] demonstrated that those specific body weight and shape considerations perceived to be crucial to control or suppress, may be impetuous threats for indulging in drunkorexia behavior. Hence, the strenuous strive to monitor calories through risky compensatory behaviors seems to be a vain cognitive approach to acquire some sense of control, recognized as lacking, on inner reactions [6]. In addition, negative metacognitive beliefs about the uncontrollability of thoughts were perceived to be crucial in drunkorexia. Specifically, pieces of research speculated that body dissatisfaction, fear of weight gain, and perpetual worrying considerations tend to become the central core of negative assessments eventually [94]. Hence, individuals undergo complex metacognitive processes leading them to perceive their inaccurate negative beliefs as unmanageable and damaging; therefore, finding themselves indulging in risky restrictive and compensatory eating patterns to apprehend the adverse outcomes such as body weight gain [56].

Finally, concerning the hypothesized indirect effects, our research has demonstrated that metacognitive beliefs about alcohol use significantly mediated the association between emotional regulation strategies and an individual's propensity to indulge in drunkorexia behavior. Our results can be interpreted in the light of previous research, and provide new evidence about the complex interplay between cognitive and emotional factors underlying drunkorexia behaviors. Previous literature has noticed that whenever emotional expressions surge, individuals attempt to manage their thinking process [51]; more specifically, to manage the cognitive manifestations of underlying struggling in regulating emotions and coping with inner discomfort [65]. A previous study has revealed that adolescents who experience negative affects and difficulties in managing their emotional responses may partake in alcohol abuse in response to metacognitive beliefs about alcohol [20]. Accordingly, our findings demonstrated the adequacy of this emotional-cognitive pattern also for explaining drunkorexia behaviors, indicating that emotion regulation processes may be associated with some alcohol-related metacognitive beliefs, which in turn prompt youths to adopt drunkorexia behavior, trusting in the functionality of drinking as a self-regulation strategy [23]. In addition, it has been found that drunkorexia may be motivated by the desire to enhance positive affect [12]; as cognitive reappraisal represents an attempt to reinterpret a situation in a more positive way, the beliefs that alcohol may help to achieve such goal, may drive individuals to use this dysfunctional behavior as a self-regulation strategy [77, 81]. Thus, believing that alcohol may be useful, for instance, to reach a more relaxed state, or to make negative thoughts or emotions more manageable and bearable, may influence and contribute to the use of reappraisal.

Furthermore, other findings have highlighted the interaction between emotional and metacognitive processes in predicting problem eating behaviors [62]. Previous studies demonstrated indeed that individuals with poor emotion regulation strategies seemed to engage in maladaptive eating behavior only when a high need to control thoughts is experienced. Conversely, if the need to control happened to be low, the shortfall of emotion regulation did not predict risky eating behavior, therefore speculating the role of metacognition as a fundamental protective factor [79]. In line with this perspective, Capobianco et al. [95] interestingly observed that metacognitive processes highly impacted personal responses to inner distress. Indeed, individuals with higher levels of emotional dysregulation are more vulnerable to the development of specific metacognitions about alcohol use, leading to an upsurge in drinking behavior.

Moreover, it has been noted that both emotion regulation strategies and metacognitive beliefs may be influenced by the control people perceive about events and situations [77, 82]; thus, the period of uncertainty and feeling of loss of control that the Covid-19 pandemic involved, in which data were collected, might have had an impact on emotional and cognitive processes. For instance, it has been showed that people are more likely to use expressive suppression if they believe that their emotions are uncontrollable, whereas they are more inclined to use cognitive reappraisal if they think that their emotions can be controlled and then modifiable [82]. Overall, our findings are generally in accordance with previous literature about both alcohol and eating behaviors, expanding the evidence of drunkorexia behaviors, and also providing new specific insights about the emotional and metacognitive processes behind them. Our study demonstrates indeed that both adaptive and maladaptive emotion regulation processes may have a significant role in activating alcohol-related metacognitive beliefs, which in turn lead young people to indulge in drunkorexia behaviors.

### Clinical implications

The present study sheds light on underlying mechanisms triggering drunkorexia behavior. Therefore, the results would contribute to further understanding of the emotional and the metacognitive processes of the risky eating pattern at an early stage of onset, providing researchers and clinicians assets for early screening for at-risk individuals, where deficient emotional strategies and lack of metacognitive skills require targeted programs and management procedures. Hence, efforts could focus on enhancing metacognitive processes for adequate monitoring of internal distress and strengthening emotional strategies to protect individuals from indulging in risky drunkorexia behavior and help them gain a sense of personal control. Furthermore, it provides a possible therapeutic path for psychologists to implement functional cognitive patterns to help individuals regain control over their maladaptive emotions and behaviors, provide them with a tool for deeper understanding of their thoughts hence, a tool to control them for their best interest.

### Limitations

The research on drunkorexia is still limited, especially in Lebanon, and therefore this research project can be considered a pioneer in the field. Indeed, very little is known about the mechanisms underlying this behavior. To the best of our knowledge, this study is also one of the first attempts in research to analyze the relation between drunkorexia, emotion regulation strategies, and alcohol-related metacognitions, providing a contribution to the interplay between emotional and cognitive processes associated with the engagement in drunkorexia. Furthermore, conversely to existing studies on this topic, which mainly included college-aged students, our study involved a sample with a wider age range. Notwithstanding, the present study is not exempt from some limitations. First, the cross-sectional design of our research does not allow the exploration of causal associations, and only correlational relationships can be inferred among study variables. Future studies should replicate our results adopting a longitudinal design to test the course and long-term outcomes of drunkorexia. Second, this research relied on self-report measures, thus, data may be subjected to inaccuracy and potential desirability bias in the responses. Third, a selection bias is possible because of the convenient sampling technique used to recruit participants; therefore, our results might not be generalizable to the whole population (especially that men and well-educated participants were more represented). A residual confounding bias is also possible since not all factors associated with drunkorexia were considered in this paper.

## Conclusion

This study demonstrated that emotional and metacognitive processes are associated with drunkorexia, addressing as well the mediating effect between deficient emotional regulation and risky behavioral patterns. Overall, our results would speculate that the lack of emotional and cognitive assets might enhance internal distress perceived as out of control, leading individuals to indulge in maladaptive behavioral patterns for managing the underlying impairment. Furthermore, this study has also highlighted a link between drunkorexia and a functional strategy of emotion regulation. Specifically, these findings suggest that when individuals are motivated to enhance positive affect and believe in the usefulness of alcohol in achieving such goal, they may prefer to use cognitive reappraisal, which may help to reframe the emotional situation more positively

## Data Availability

The datasets generated and/or analyzed during the current study are not publicly available due to the policies of the ethics committee, but are available from the corresponding author on reasonable request.
